# Functional and genomic insights into BHET-degrading *Stenotrophomonas* sp. isolated from the marine plastisphere

**DOI:** 10.3389/fmicb.2025.1680692

**Published:** 2025-10-23

**Authors:** Ye Zhuo, Chun-Zhi Jin, Chang-Soo Lee, Hyung-Gwan Lee

**Affiliations:** ^1^Cell Factory Research Center, Korea Research Institute of Bioscience and Biotechnology (KRIBB), Daejeon, Republic of Korea; ^2^Department of Biotechnology, KRIBB School of Bioscience, Korea University of Science and Technology (UST), Daejeon, Republic of Korea; ^3^Fungi Research Division, Microbial Research Department, Nakdonggang National Institute of Biological Resources, Sangju, Republic of Korea

**Keywords:** plastisphere, BHET biodegradation, *Stenotrophomonas*, BHETase, TPA transporter

## Abstract

Enzymatic degradation of polyethylene terephthalate (PET) has dramatically advanced through protein engineering of PETase, accelerating the biocatalytic depolymerization process. However, the subsequent microbial valorization of PET-derived intermediates, such as bis (2-hydroxyethyl) terephthalate (BHET) and terephthalic acid (TPA), remains limited because of restricted availability and suboptimal activity of specific biocatalytic enzymes. In this study, eight microbial species were isolated from the enriched cultures of marine plastic waste, using 1% PET, BHET and TPA as the sole carbon source. *Stenotrophomonas* was the only species detected in all the cultures. Strain WED208 was isolated from a BHET-enriched culture and selected for its potential role in plastic degradation. Phylogenetic analysis based on the 16S rRNA gene revealed 99.4% similarity to *Stenotrophomonas riyadhensis LMG 33162T*; however, it exhibited distinct physiological and genomic features. Strain WED208 degraded approximately 30% of BHET into mono (2-hydroxyethyl) terephthalate (MHET) over 30 days but did not catalyze further conversion to TPA. Comparative analysis identified a putative BHETase (WED208_02958) containing conserved catalytic residues (Ser90, Asp217, and His245). Structural modeling and protein-ligand docking analysis confirmed a key interaction between Ser90 and the ester bond of BHET, supporting the microbial hydrolytic function. Although strain WED208 could degrade neither PET nor TPA, its genome harbored two putative PETase genes and key enzymes potentially involved in TPA degradation, including diol dehydrogenase (*tphB*), MFS transporter (*pcaK*), and LysR-type transcriptional regulator (*lysR*). These findings suggest that WED208 is a promising microbial resource for enzyme engineering and has potential use in microbial consortia to enhance PET biodegradation and upcycling.

## 1 Introduction

Plastics have become essential in modern society owing to their versatility, cost-effectiveness, and durability. Global production is projected to reach 800 million and 1.6 billion tons by 2035 and 2050, respectively (Ali et al., 2021). This rapid growth has resulted in serious environmental problems, particularly plastic waste accumulation, which threatens public health and ecological stability (Kumar et al., 2021; Salinas et al., 2025). Therefore, innovative and sustainable solutions are urgently required to mitigate the effects of plastic pollution. PET is extensively used in automobile tire reinforcement, food packaging, and building materials (Kotova et al., 2021). Despite its widespread use, improper disposal and incineration of PET pose serious environmental and health hazards (Baek et al., 2022; Hussain et al., 2023). The growing dependence on PET-based products, combined with inappropriate waste management systems, has made PET pollution a global concern. Therefore, developing sustainable methods for PET degradation or recycling is essential to minimize environmental and health impact.

Biodegradation is a promising approach for reducing PET pollution. Several microorganisms, including *Ideonella sakaiensis*, *Saccharomonospora viridis*, *Pseudomonas putida,* and *Stenotrophomonas maltophilia*, showed significant PET degradation capabilities (Miyakawa et al., 2015; Yoshida et al., 2016; Vague et al., 2019; Din et al., 2023). These microbes produce enzymes including lipases, cutinase, and esterases (Eberl et al., 2009), which catalyze the hydrolysis of PET into intermediate compounds such as bis (2-hydroxyethyl) terephthalate (BHET), mono (2-hydroxyethyl) terephthalate (MHET), terephthalic acid (TPA), and ethylene glycol (EG) (Kim et al., 2022). Among these, BHET is particularly notable because of its structural similarity to PET and its potential role in microbial degradation pathways (Joo et al., 2018). BHET biodegradation mechanism study could provide crucial insights into understanding its toxicity to humans and environmental impacts. Moreover, the use of BHET-degrading microorganisms as whole-cell biocatalysts offers several advantages, including the reduction of the inhibitory effects on PET hydrolases and minimization of the toxicity of degradation byproducts such as TPA (Qiu et al., 2020). While extensive research has focused on the microbial degradation of PET products, such as TPA and EG, few studies have explored microorganisms capable of utilizing BHET as the sole carbon source, which could contribute to the improvement of bioremediation strategies.

Plastispheres have been extensively investigated as novel bioresources for the biodegradation of plastic. Over extended periods, microbial consortia naturally attach to plastic debris in the environment and in adhesion, biodeterioration, biofragmentation, assimilation, and mineralization. NGS technology allows us to understand the distinct dominant taxa between the plastisphere and normal microbial habitats (Li et al., 2024). Based on these findings, co-cultures of plastic-degrading or associated microbes have been used to enhance plastic biodegradation and upcycling. For instance, Schaerer et al. identified *Hydrogenophaga* as an aromatic compound degrader and *Paracoccus* as a specialist in terephthalamide degradation using metagenomic and metatranscriptomic approaches (Schaerer et al., 2023). Furthermore, integrating mixed microbial consortia with pretreatment processes improves the biodegradation efficiency of polyolefin plastics (Lomwongsopon et al., 2025).

In this study, we aimed to isolate plastic-associated bacteria from the marine plastisphere (specifically from waste plastics collected from the coast) and investigate their potential roles in plastic degradation through substrate assimilation experiments. We further explored the taxonomic position of the selected isolate based on its physiological and genomic characteristics and identified key enzymes involved in plastic biodegradation using comprehensive genomic analysis, structural modeling, and protein-ligand docking analysis. Eight distinct bacterial species were isolated from marine plastic waste, and *Stenotrophomonas* sp. WED208 was selected for its potential role in the degradation of PET and its intermediates, BHET and TPA, respectively. This study proposes strain WED208 as a promising microbial resource for sustainable plastic waste management, with potential applications in enhancing enzymatic efficiency through protein engineering and elucidating the regulatory mechanisms underlying plastic degradation pathways.

## 2 Materials and methods

### 2.1 Strains and culturing conditions

To explore the microorganisms that decompose marine microplastics, 63 pieces of plastic waste (plastic bottles, caps, straws, and plastic films) that were either discarded or partially buried in sediment or among rocks were collected from eight shoreline locations around Jeju Island. The collected samples were stored at 4 °C and transferred to the laboratory for analysis. For plastic waste-associated bacterial isolation, plastic samples were initially enriched in R2A broth medium for 1 week. The enriched cultures were subsequently inoculated into a modified R2A medium (lacking glucose and starch) supplemented with 1% (w/v) plastic powder (PET, BHET, or TPA) and incubated for 4 weeks. PET powder (100–200 um) was prepared by Dr. Jeong (KRIBB, Korea) and BHET and TPA were purchased from Sigma-Aldrich (United States). Finally, the cultures were spread onto R2A agar plates to isolate individual bacterial colonies (Supplementary Figure S1). The isolates were stored in a 20% v/v glycerol solution at −80 °C.

### 2.2 16S rRNA gene sequencing and phylogenetic analysis

A partial 16S rRNA sequence of the strain was amplified using the universal primers 27F and 1492R for phylogenetic analysis (Jin et al., 2022). The 16S rRNA sequences of closely related microbes were retrieved from the EzBioCloud database.^1^ A phylogenetic tree was constructed using the neighbor-joining method with the Kimura 2-parameter model (Tamura et al., 2021) in MEGA 11.

### 2.3 Phenotypic and physiological characterization

R2A medium was used to assess the morphological, physiological, and biochemical characteristics of the strain. Growth was evaluated at different temperatures (4–45 °C), salt concentration [0–12% (w/v) NaCl], and pH values (4–12) for 3–5 days under aerobic conditions. Antibiotic susceptibility assays were performed using filter paper disks containing 14 different antibiotics (Table 1). Additional physiological characteristics were determined using API 20NE and API ZYM (bioMérieux) according to the manufacturer’s instructions.

**Table 1:** Phenotypic and physiological characteristics of *Stenotrophomonas* sp. WED208.

Characteristics	WED208
Isolation source	Enrichment culture (Plastic waste from the coast near Seoubong peak in Jeju island)
Oxidase	−
Growth at/with:	
Medium	Bennet, LB, ISP2/4, PDA, R2A, NA, MA
Temperature	10–40, 28
NaCl (%)	0–8, 0
pH	5.0–12.0, 7.0
Hydrolysis of:	
Esculin (β-glucosidase activity)	+
Gelatin (protease activity)	+
β-galactosidase	+
Assimilation of:	
Maltose	+
D-Mannose	+
N-Acetyl glucosamine	+
Malic acid	+
Trisodium citrate	+
Enzyme activities	
Cystine arylamidase	+
α-chymotrypasin	+
α/β-galactosidase	+
β-glucuronidase	+
N-Acetyl-β-glucosaminidase	+
α-mannosidase	+
α-fucosidase	+
Antibiotic resistance (diameter/mm)	
Nalidixic acid (30)	24
Tetracycline (30)	21
Amikacin (30)	12
Ampicilin/Sulbactam (20)	−
Kanamycin (30)	11
Vancomycin (30)	10
Chloramphenicol (30)	20
Teicoplanin (30)	8
Streptomycin (25)	12
Gentamicin (30)	12
Spectinomycin (25)	−
Rifampicin (30)	22
Lincomycin (15)	−
Erythromycin (30)	−

### 2.4 Whole-genome sequencing and assembly

A genome sequencing library was prepared using the TruSeq Nano DNA Kit, generating paired-end reads of 2 × 151 bp and was sequenced using the Illumina HiSeq 2500 platform. Raw sequence data were quality-checked using FastQC v. 0.11.5,^2^ and adapter sequences and low-quality reads were trimmed using Trimmomatic v0.36 (Bolger et al., 2014). Quality-filtered reads were assembled *de novo* using SPAdes v3.15.0 (Bankevich et al., 2012). Assembly quality was evaluated using BUSCO v5.1.3 (Simão et al., 2015). Genome coverage and size were estimated using Jellyfish v2.2.10 (Marçais and Kingsford, 2011) and GenomeScope (Vurture et al., 2017). Predicted coding sequences (CDS) were identified using Prodigal, and functional annotation was perfomred using Prokka (Seemann, 2014). BLAST^3^ was used for genome sequence comparisons.

### 2.5 Genome annotation and comparative study

Rapid Annotation using Subsystem Technology (RAST) and the evolutionary genealogy of genes (EggNOG) were used for gene prediction (Brettin et al., 2015). PlasticDB (V7.0) was used to identify the proteins associated with plastic biodegradation (Blin et al., 2023). A phylogenomic tree was constructed using the Genome BLAST Distance Phylogeny method on the TYGS platform (Meier-Kolthoff and Göker, 2019) and visualized using the web-based software iTOL v7.^4^ The Bacterial and Viral Bioinformatics Resource Centers (BV-BRC) were analyzed using the default settings. Antibiotic-resistance genes were identified using the Comprehensive Antibiotic Resistance Database.^5^ Circular genome maps were generated using Proksee^6^ (Grant et al., 2023).

Genomic data were obtained from the National Center for Biotechnology Information (NCBI), and the accession numbers are listed in Supplementary Table S1. For the pan-genome analysis, eight strains closely related to strain WED208 were selected using the Anvi’o 8 pan-genomic workflow (Delmont and Eren, 2018; Eren et al., 2020). Genes were clustered in Anvi’o using BLASTP from NCBI with the default settings.

### 2.6 BHET degradation assay

The culture medium was R2A broth supplemented with 1% (w/v) BHET powder. After thorough mixing, the culture medium was inoculated with a 1% (v/v) bacterial solution (A_600_ = 0.4). All experimental samples were prepared in triplicates, along with three negative controls that did not contain bacteria. The cultures were incubated at 28 °C for 30 days, and bacterial growth (A_600_) and pH were measured every day. The culture solution was collected every 5 days, and the supernatant was concentrated. Methanol was added to prepare a 1 mg/mL solution, and the degradation activity was analyzed by HPLC after filtration through 0.20 μm.

HPLC analysis was performed using an Agilent Technologies 1260 Series system equipped with an AccucoreTM XL C18 (4.6 × 250 mm 4 μm) column (Agilent Technologies, Santa Clara, CA, United States). The mobile phase consisted of 50 mM PBS (solvent A) and methanol (solvent B) at a flow rate of 0.5 mL/min and a column temperature of 40 °C. The gradient was set as follows (all percentages were v/v): starting with 80% solvent A and changing to 60% solvent A at 12 min. The injection volume was 10 μL. Pure BHET, MHET, and TPA solutions (dissolved in methanol) were used as references.

### 2.7 Homology alignment and docking

Potential homologous hydrolases of LCC, TfCut2, PET40, IsPETase, TfH, and ScLipA were identified by performing BLASTp search of the strain WED208 genome using the default settings. The hits were ranked based on the BLAST score, query cover, percent identity, and the presence of the catalytic triad and substrate-binding site. The similarity of the protease genes between strain WED208 and the other strains was compared with the gene sequences in the NCBI database.

The 3D structure of WED208_MEX5405504.1 was predicted using AlphaFold 3, supported by the AlphaFold Server (Abramson et al., 2024). Molecular docking was performed with SwissDock (Eberhardt et al., 2021; Bugnon et al., 2024), and model visualization and editing were conducted using ChimeraX 1.8 (Meng et al., 2023).

### 2.8 TPA transporter proteins

To predict the distribution of TPA transporters in the strain WED208, we constructed a database of homologous proteins from public sources and aligned the WED208 coding sequence (CDS) with it to identify candidate transporters. For efficient and accurate sequence alignment, we used the command-line version of Diamond, which is known for its speed and precision (Buchfink et al., 2021). The following parameters were set during the alignment: the expected cutoff value (*E* value) was 10^−5^ to ensure significant results, a minimum alignment length of 30 amino acids to ensure reliability, and a minimum similarity percentage of 50% to ensure sufficient homology among aligned proteins.

## 3 Results

### 3.1 Phylogenetic and physiological characterization

More than 25 distinct colonies were selected based on their morphological characteristics and identified by 16 s rRNA sequencing. These isolates belonged to four genus, *Stenotrophomonas*, *Bacillus*, *Pseudomonas,* and *Raoultella,* with *S. riyadhensis* being consistently enriched across all substrates. Among the identified species, *Stenotrophomonas* was the only genus found in all enrichment cultures, suggesting its potential as a unique candidate for further studies on the biodegradation of PET and its intermediates. Although nine strains belonged to the genus *Stenotrophomonas*, they shared identical 16 s rRNA sequences. Consequently, strain WED208, isolated from the BHET enrichment culture, was selected for further analysis.

**Figure 1:**
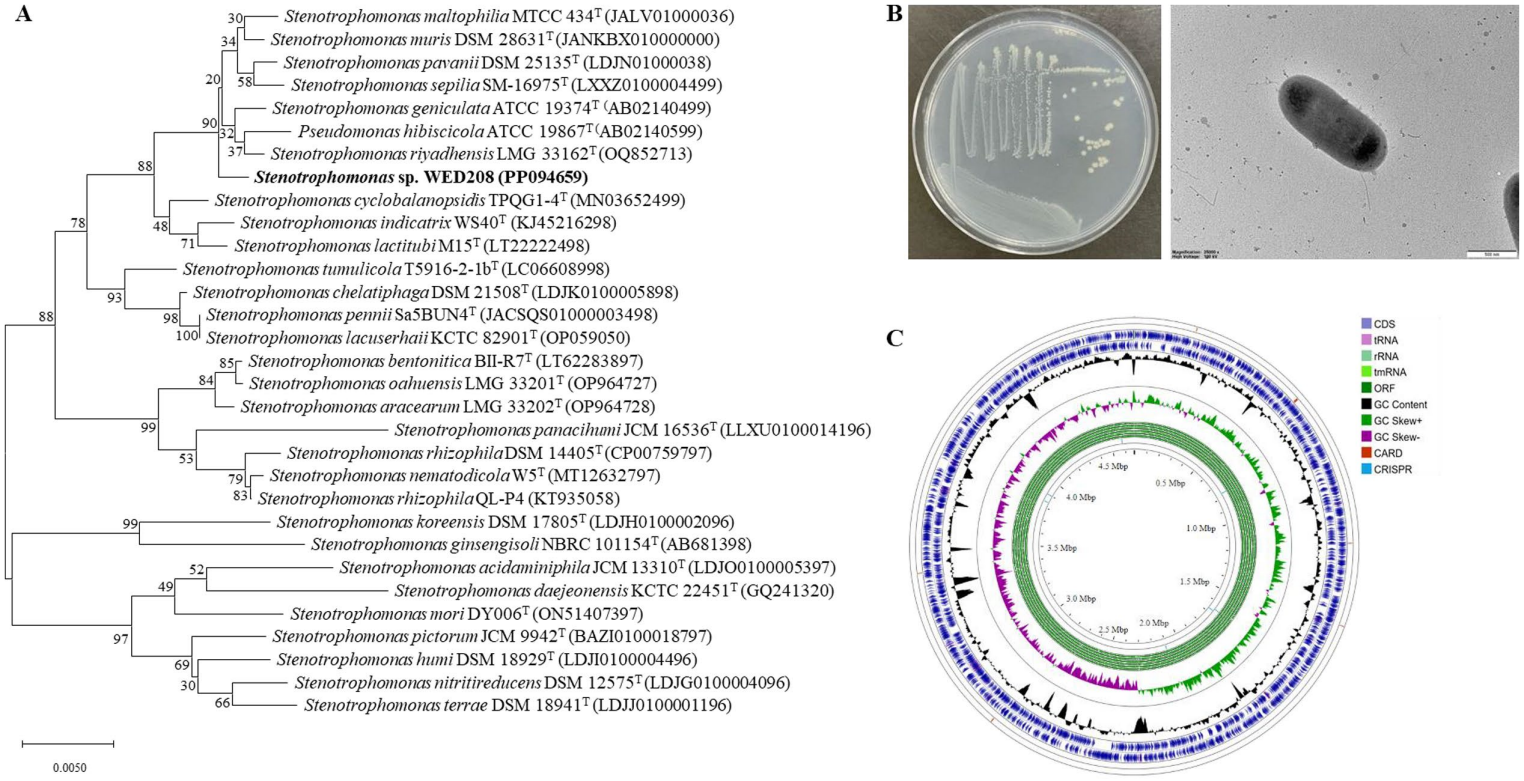
Phylogenetic tree, morphological characterization, and genome map of strain WED208. **(A)** Neighbor-joining tree of strain WED208 based on 16S rRNA gene sequences. Bootstrap values >50% are shown at the branch points. Bar: 1 nt substitution per 100 nucleotides. **(B)** Cultivation photo and scanning electron microscopy image of WED208. **(C)** Circular graphic showing the distribution of genome annotations in strain WED208.

The 16S rRNA gene sequence of strain WED208 was uploaded to the EzBioCloud database for comparison. It exhibited the highest identity to *S. riyadhensis* LMG 33162^T^ (99.82%), *S. pavanii* DSM 25135^T^ (99.36%), and *S. geniculata* ATCC 19374^T^ (99.36%), followed by *S. maltophilia* KACC 11358^T^ (99.29%) and *S. hibiscicola* ATCC 19867^T^ (99.15%) (Figure 1A). Despite its close phylogenetic relationship with *Stenotrophomonas* species, strain WED208 displayed distinct genotypic characteristics, including genome size, variation in the number of annotated genes, and the number and distribution of accessory and singleton genes.

Cells of strain WED208 were non-spore-forming, rod-shaped, Gram-negative, and measured 1.3 × 0.6 μm (Figure 1B). After 3 days of cultivation at 28 °C, the strain formed pale yellow, smooth, glossy, and spherical colonies on the medium. The phenotypic and physiological characteristics of strain WED208 are summarized in Table 1.

### 3.2 Genomic features

Strain WED208 possessed a 4,637,711 bp circular chromosome with a GC content of 66.5%. Genes were identified through genome annotation using Prokka, including 4,176 protein-coding genes (CDSs), 73 tRNA genes, 3 rRNA genes, and 1 tmRNA gene (Supplementary Table S1). A circular genome map was generated using the Proksee Server to illustrate the distribution of these genome annotations (Figure 1C). Functional annotation of strain WED208 was performed using RAST and EggNOG, and the results showed that the strain contained 305 subsystems across 24 functional categories (1,479 genes) and 22 functional clusters (2,998 genes; Supplementary Figure S2). Notably, WED208 contained 23 and 146 genes associated with aromatic compound metabolism and membrane transport, respectively. Several candidate enzymes with potential plastic degradation activity were identified, including 32 esterases, 6 lipases, and 13 α/β-hydrolases involved in ester bond hydrolysis.

**Figure 2:**
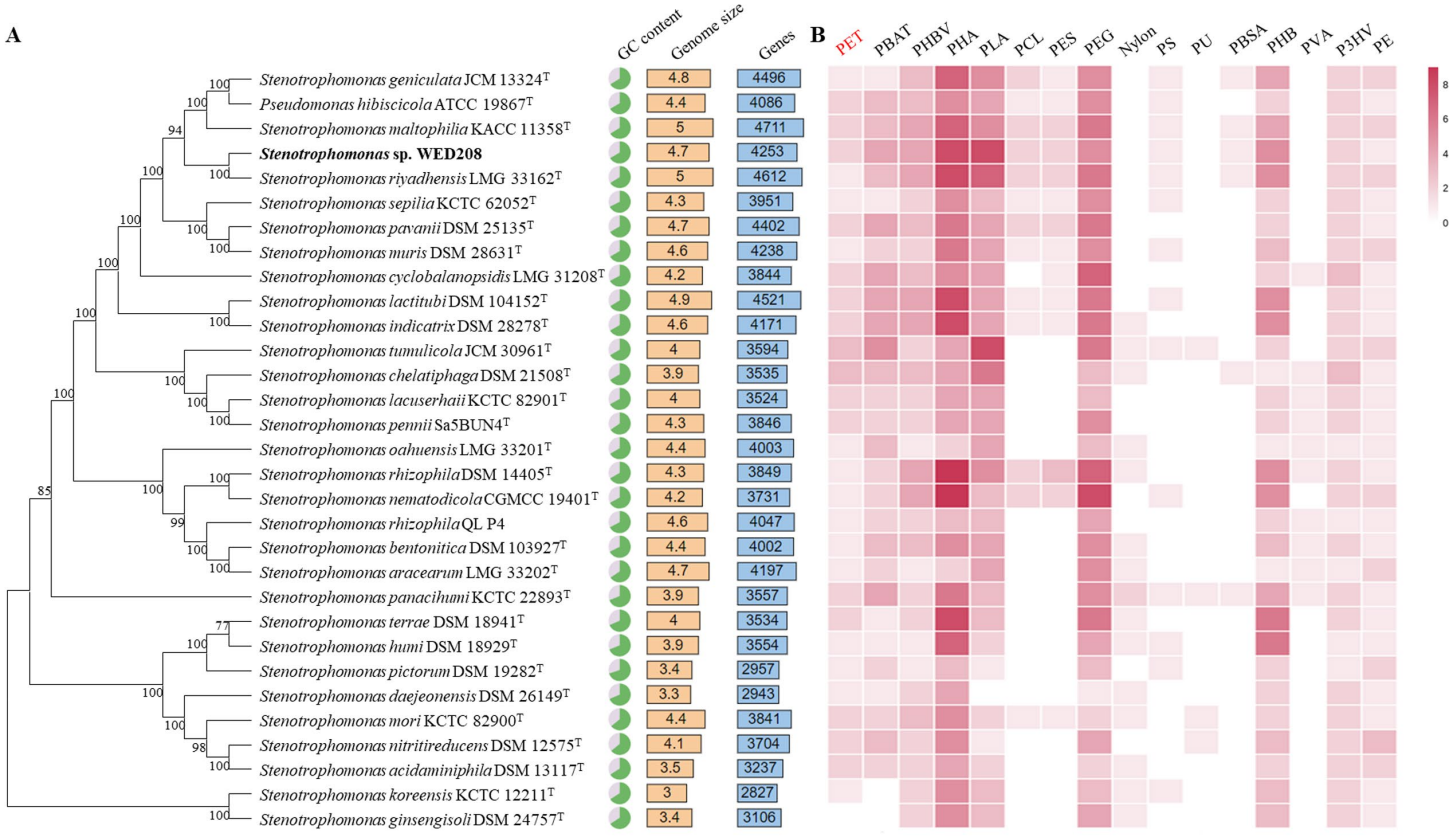
Phylogenomic tree and annotations based on the genome sequences. **(A)** Genomic tree of WED208 and related species belonging to *Stenotrophomonas* through TYGS. The output was visualized using the iTOL. **(B)** Annotation of plastic degradation related gene from the genus *Stenotrophomonas* based on PlasticDB. The number of annotation types is represented by the color gradient.

The taxonomic position of strain WED208 within the genus *Stenotrophomonas* was also confirmed through phylogenomic analysis with other 31 *Stenotrophomonas* species. Figure 2A summarizes the genome size, gene count, and GC content of *Stenotrophomonas* species. A comprehensive pan-genomic analysis of nine strains within the clade revealed 7,385 gene clusters (37,855 genes), including 2,676 core gene clusters, 2,339 accessory gene clusters, and 2,370 singleton gene clusters (Supplementary Figure S3). Although strain WED208 shares the conserved functional backbone of the genus, its unique accessory and singleton genes may provide adaptive advantages and novel metabolic pathways, making it a promising candidate for further investigation into biodegradation and environmental biotechnology applications. Analysis of antibiotic resistance genes in strain WED208 was summarized in Supplementary Tables S1, S2 that revealed multiple resistance-related genes and their respective mechanisms (Garneau-Tsodikova and Labby, 2016).

### 3.3 Biodegradation of BHET and TPA

Biodegradation activity was assessed by analyzing the degraded intermediates. Although *Stenotrophomonas* was initially isolated from enrichment cultures containing PET, BHET, or TPA, strain WED208 only degraded BHET. No intermediates (BHET, MHET, and TPA) were detected in the PET culture, and no reduction in TPA was observed in the TPA culture. In contrast, in the BHET culture, BHET levels decreased, whereas MHET levels increased (Supplementary Figure S4).

**Figure 3:**
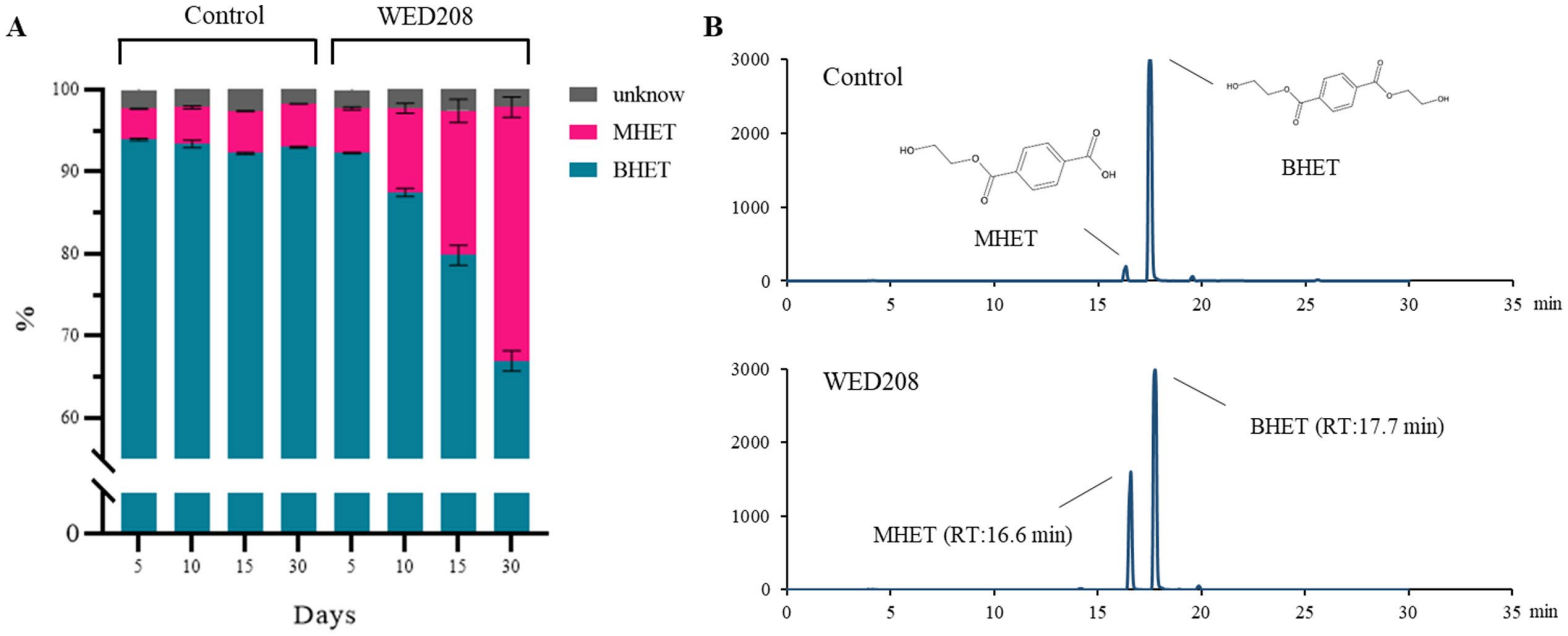
Strain WED208 degrades BHET to produce MHET in liquid culture (*n* = 3). **(A)** Control sample containing modified R2A medium and BHET (1%). The areas represent the percentage of each compound in the culture. BHET, MHET, and unknown compounds are represented by cyan, magenta, and gray, respectively. **(B)** HPLC profiles of BHET and MHET in both control and test samples after 30 days.

Growth was monitored for 30 days by measuring OD and pH daily. BHET degradation was analyzed using HPLC on days 5, 10, 15, and 30. Although the initial growth rate was low, it progressively increased over time, with BHET conversion closely correlated with cell growth (Supplementary Figure S5). In the strain WED208 cultures, BHET degradation primarily led to MHET accumulation rather than TPA. The BHET reduction increased gradually, reaching approximately 30% by day 30 (Figure 3). Some BHET degradation was also observed in the control, likely due to the instability caused by UV sterilization before cultivation. However, the BHET reduction in the strain WED208 culture was significantly higher than that in the control. No TPA accumulation was detected, indicating that the strain WED208 hydrolyzed BHET to MHET but did not further degrade MHET to TPA.

### 3.4 Identification of potential BHETase and TPAase

Plastic degradation genes in the whole-genome sequence of strain WED208 were investigated using PlasticDB. The strain WED208 harbors several enzymes potentially involved in degrading various plastics, including PET, PBAT, PHBV, PHA, PLA, PEG, and PHB (Figure 2B). These enzymes include PETases, esterases, lipases, proteases, carboxylesterases, dehydrogenases, and depolymerases. During PET degradation, these enzymes typically catalyze the hydrolysis of ester bonds, breaking PET into smaller molecules, such as BHET and TPA, which are more readily assimilated by microorganisms (Lu et al., 2022). Although WED208_00500 and WED208_02014 were annotated as esterase and PETase and linked to PET degradation, no degradation products were observed in cultures containing 1% PET powder, suggesting that these enzymes may require specific conditions or cofactors for activation.

**Figure 4:**
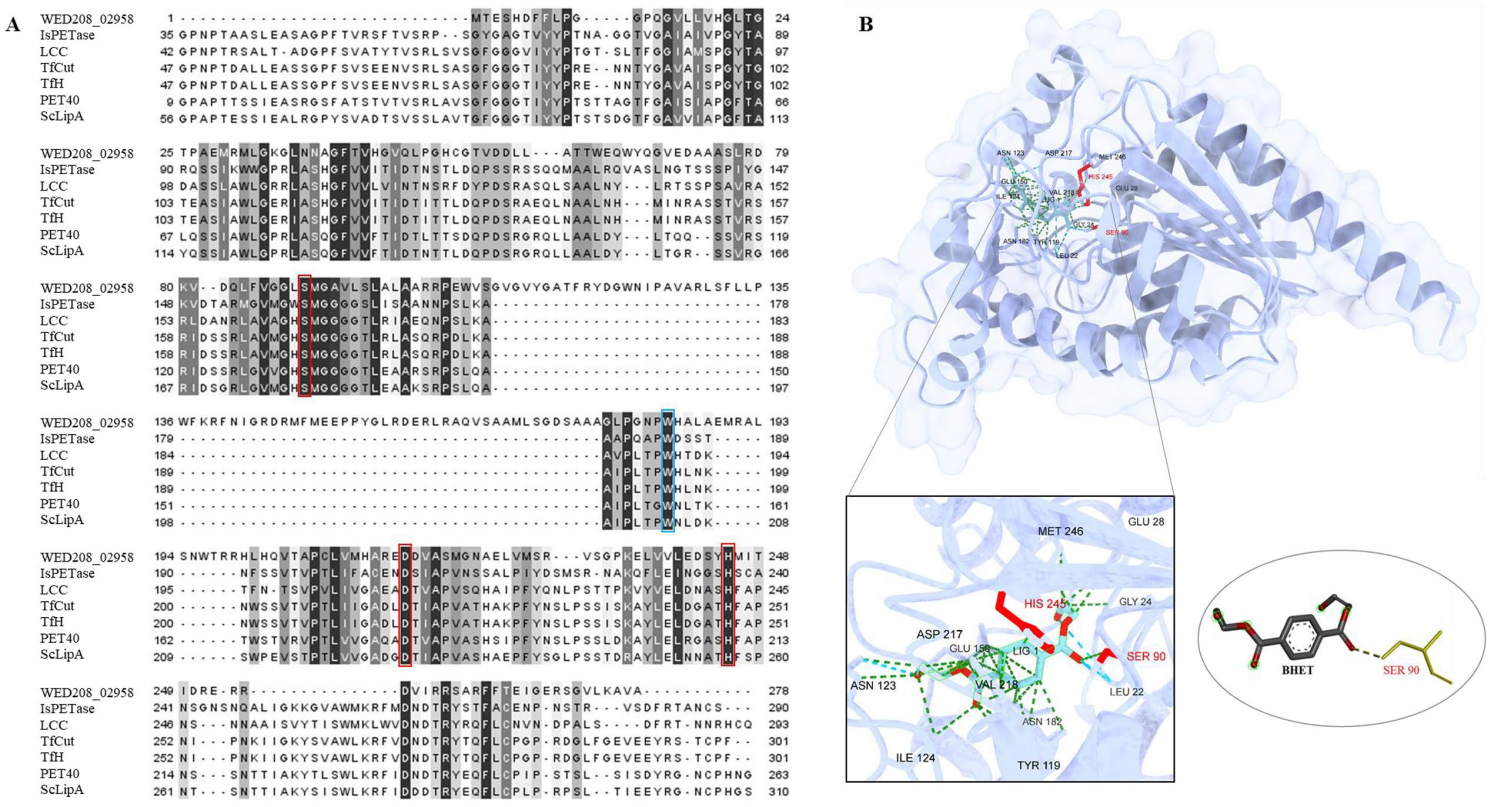
**(A)** Sequence comparison of WED208_02958 with LCC (G9BY5744), TfCut2 (Q6A0I425), PET40 (WAU86704.128), IsPETase (A0A0K8P6T729), TfH (WP_011291330.1), and ScLipA (Q9L2J6), with the binding site shown in blue boxes and the catalytic triad highlighted in red boxes. The percent identity is shown after alignment. **(B)** Structure prediction model of WED208_02958 built using AlphaFold and docked with the BHET ligand. The relevant amino acid residues are enlarged and shown, among which SER90 is the key residue for catalyzing ester bond formation.

To explore the potential role of the strain WED208 in BHET metabolism, a Protein BLAST search was conducted using several BHETases/PETase enzymes, such as LCC, *Tf*Cut2, *Tf*H, PET40, *Is*PETase, and *Sc*LipA. WED208_MEX5405504.1 retains conserved functional domains essential for BHET hydrolysis. Sequence alignment (Figure 4A) revealed a low overall similarity between WED208_MEX5405504.1 and the six BHETase/PETase enzymes, attributed to insertions (positions 109–177) and deletions (positions 1–29). These structural differences may explain the variations in the sequence profiles. Despite these variations, WED208_MEX5405504.1 retained the key residues essential for BHETase activity. The catalytic triad, consisting of serine (Ser90), aspartate (Asp217), and histidine (His245), is crucial for hydrolyzing the ester bond in BHET. Tryptophan (Trp184), a binding-site residue involved in substrate recognition, is also conserved. These findings suggest that WED208_MEX5405504.1 possesses BHETase activity and may contribute to the BHET metabolism.

To further investigate the functional conservation of WED208_MEX5405504.1, a protein docking analysis (Figure 4B) was performed using BHET as the ligand. The analysis revealed that BHET binds to the active site of WED208_MEX5405504.1, where ten amino acid residues interact with it. Notably, Ser90 interacted with one of the ester bonds of BHET, thereby facilitating its hydrolysis. This supports the known catalytic mechanism of BHETase, in which the active-site serine acts as a nucleophile to cleave the ester bond, leading to BHET degradation.

## 4 Discussion

The genus *Stenotrophomonas* is widely recognized for its role in bioremediation, particularly in the degradation of various pollutants, including xenobiotics, oils, dyes, and polycyclic aromatic hydrocarbons (Coleman et al., 2002; Arulazhagan et al., 2017; Alvarado-Gutiérrez et al., 2020). Recently, the plastic-degrading potential of *Stenotrophomonas* species has garnered increasing attention. These bacteria secrete enzymes, such as esterases and lipases, which break down plastic polymers into smaller, more biodegradable molecules (Table 1). *S. maltophilia* reportedly degrades PET plastics down to TPA, BHET, and MHET using cutinase-like enzymes (Din et al., 2023). Furthermore, *Stenotrophomonas* strains can degrade polyethylene and polystyrene using multiple enzymes (Peixoto et al., 2017; Xiang et al., 2023). This study demonstrated the ability of *Stenotrophomonas* to degrade BHET, providing complementary information on its known degradation capabilities.

Strain WED208, isolated from waste plastics collected from the seashore, could utilize BHET as the sole carbon source and convert it into MHET. Genomic analysis revealed the presence of a potential BHET-degrading enzyme, WED208_MEX5405504.1, a putative BHETase. Sequence conservation analysis and protein-ligand docking identified conserved catalytic residues (Ser90, Asp216, and His245), and Ser90 was found to interact with the ester bond of BHET. This is consistent with the typical serine hydrolase mechanism, in which serine acts as a nucleophile and histidine functions as a general acid/base, supported by aspartate. Despite sequence variations compared to several BHETase/PETase enzymes, WED208_MEX5405504.1 retained the essential active site residues. However, its binding affinity for BHET (−5.771 kcal/mol) is weaker than those of proteins with higher affinities (−6 to −8 kcal/mol), suggesting a reduced catalytic efficiency (Hong et al., 2024; Longarini and Matić, 2024). A stronger binding affinity typically correlates with a higher catalytic activity (Hong et al., 2024). These findings suggest that the lower binding affinity of the enzyme may limit its efficiency in BHET degradation. Recent advances in mechanism-guided enzyme engineering, such as modifications to the barrier regions of two BHETases, ChryBHETase and BsEst, have enhanced BHET accessibility and catalytic efficiency by optimizing key structural parameters using docking analysis (Li et al., 2023). The optimized variants, ΔBsEst and ΔChryBHETase, in which the barrier regions were removed and the top-scoring modifications were selected, exhibited a 3.5-fold increase in their catalytic efficiency. Applying similar engineering strategies to WED208_MEX5405504.1 may improve its catalytic activity by enhancing its interaction with BHET.

**Figure 5:**
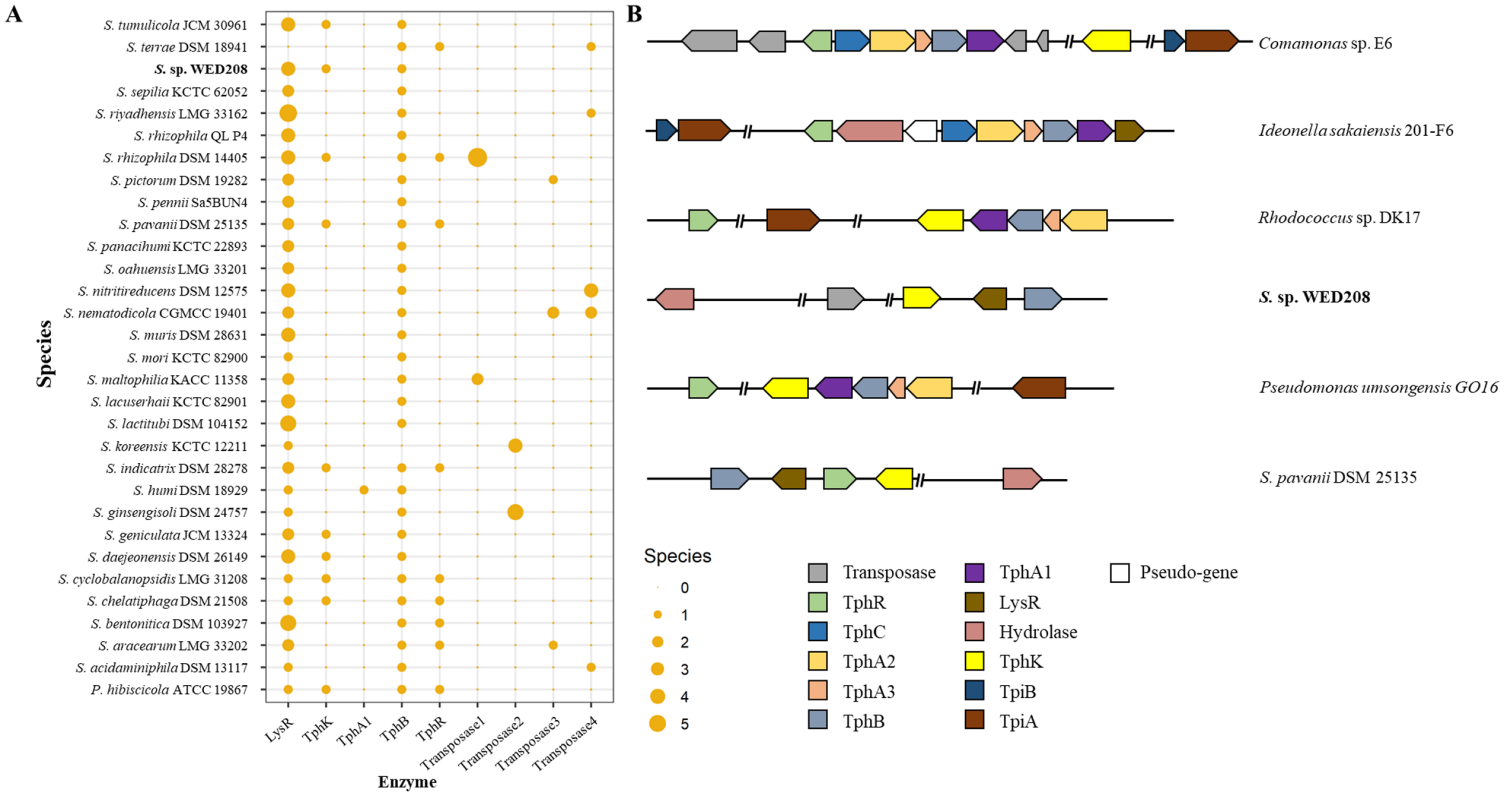
Distribution TPA catabolism-related genes in *Stenotrophomonas* genus **(A)** and schematic representation of the representative TPA metabolism gene cluster **(B)**. The upper two strains represent the tripartite tricarboxylate transporter (TTT) system, whereas the lower four strains represent the major facilitator superfamily (MFS) transporter system in diverse bacterial species. TphR (*tph* operon regulator), TphC (periplasmic solute-binding protein), TphA1A2A3 (TPA 1,2-dioxygenase), TphB (diol dehydrogenase), LysR (putative TPA regulation gene), and PcaK (MFS transporter).

TPA, an aromatic compound derived from PET, must be completely degraded to achieve effective PET depolymerization and valorization. TPA is converted to protocatechuate (PCA) via DCD, catalyzed by *tphA1A2A3* and *tphB*, and subsequently funneled into central metabolism for the production of value-added compounds, such as muconic acid, vanillic acid and pyrogallol (Kim et al., 2019). To explore this pathway in strain WED208, a comparative genomic analysis with related *Stenotrophomonas* species was conducted (Figure 5). *tphB* was conserved across all strains except *S. koreensis* KCTC 12211, whereas *tphA* was present only in *S. humi* DSM 18929. The *lysR* gene, a LysR-type regulator frequently associated with TPA clusters, was identified in all strains except *S. terrae* DSM 18941. Notably, only 10 strains, including strain WED208, carried *tphK*, a homolog of pcaK that encodes a p-hydroxybenzoic acid transporter involved in the uptake of aromatic compounds (Nichols and Harwood, 1997). In WED208, tphK is located within the predicted TPA cluster and encodes a major facilitator superfamily (MFS) transporter, consistent with MFS-type TPA transporters in *Rhodococcus* sp. and *Paraburkholderia xenovorans*. In contrast, *Comamonas* sp. employs a tripartite tricarboxylate transporter (TTT; tphC–tpiAB) that is positioned separately from the TPA operon (Hosaka et al., 2013; Yoshida et al., 2016; Gautom et al., 2021). These observations suggest that *tphK* in WED208 may function as a transporter of TPA and related aromatics.

**Figure 6:**
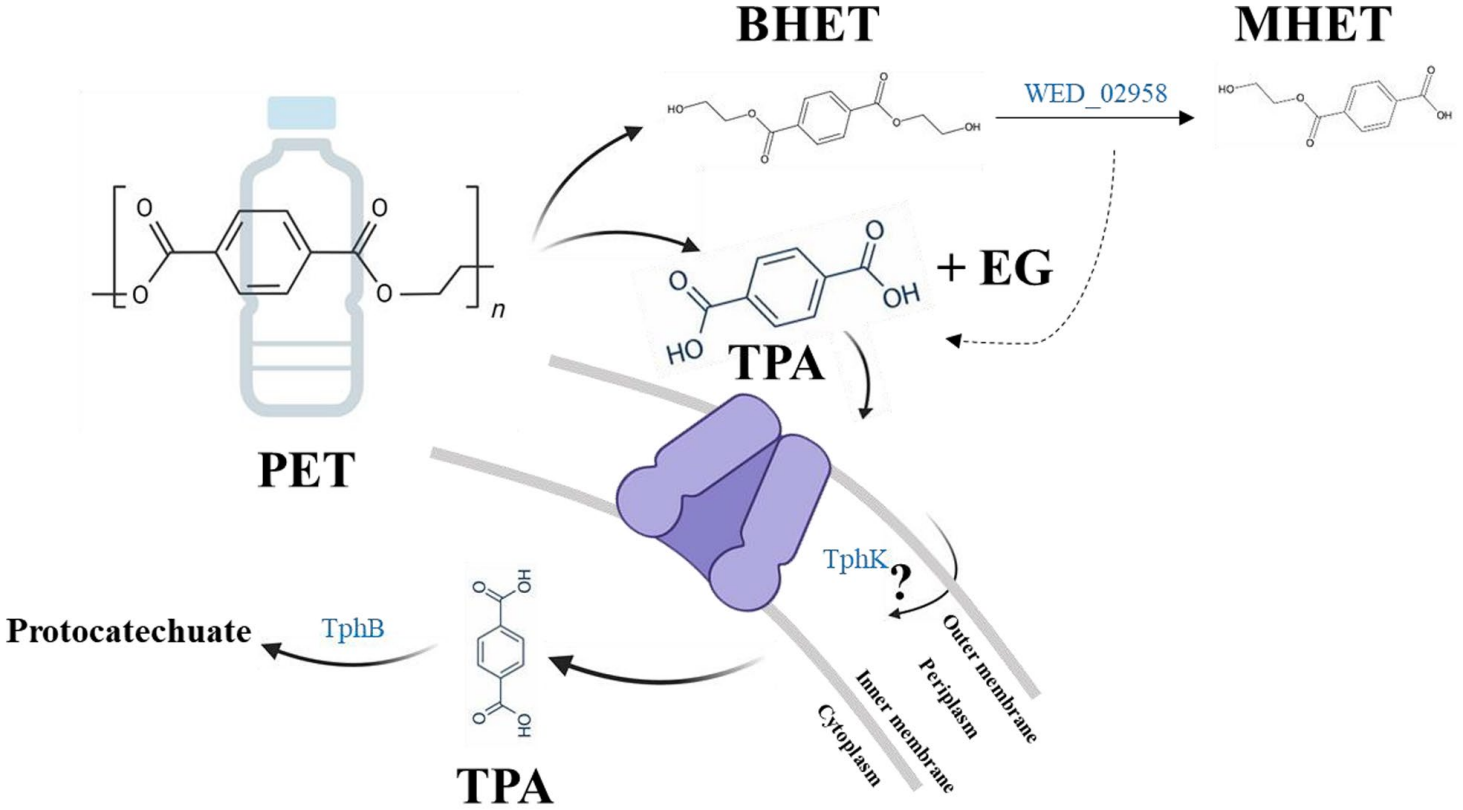
Hypothetical BHET/PET degradation pathway in strain WED208, illustrating the predicted enzyme candidates (BHETase, TphK, and TphB). PET and TPA are included to indicate the potential roles of these candidate genes, although no experimental PET or TPA degradation was observed.

Although strain WED208 lacks *tphA* and cannot complete TPA degradation, its accessory genes (*tphB, tphK,* and *lysR*) remain valuable for PET upcycling strategies. For instance, Werner et al. (2021) engineered *Pseudomonas putida* to convert TPA to β-ketoadipic acid by introducing *tphAB* from *Comamonas camanonas* and *tphK* from *Rhodococcus jostii*, while deleting *pcaiJ*, which encodes 3-oxoadipate CoA-transferase. Furthermore, identifying new TPA transporters or mechanisms is crucial for advancing microbial engineering in PET upcycling. The muconate transporter *MucK* in *Acinetobacter baylyi* ADP1 has been proposed to facilitate TPA import, potentially functioning similarly to TphK (Pardo et al., 2020). Further functional studies of tphK in the strain WED208 could expand its application in engineering microbes for PET bioconversion. Based on genomic predictions, the proposed BHET/PET degradation pathway for the strain WED208 is illustrated in Figure 6.

This study provides valuable insights into the molecular mechanisms underlying BHET degradation by *Stenotrophomonas* sp. WED208, providing a foundation for future research on its application in plastic biodegradation and upcycling. Although BHET biodegradation by the strain WED208 was slow and incomplete, these limitations could be improved through microbial consortia, which synergistically degrade plastics. Notably, eight other species were isolated from the enriched cultures derived from the same marine plastic waste samples. These isolates highlight the potential for developing microbial consortia for PET degradation. Recent research has focused on constructing plastisphere microbial communities to enhance the natural degradation of banned microplastics for bioremediation. Even if individual microbes lack enzymes associated with PET degradation, microbial consortia can compensate for these deficiencies by supporting the metabolic functions of each other, leading to enhanced degradation through synergistic interactions. Further studies are required to explore the relationship between the isolated strains and their collective potential for PET biodegradation. In parallel, enhancing the enzymatic activity of key-degrading enzymes through protein engineering and exploring the regulatory mechanisms controlling these metabolic pathways are crucial. Integrating these insights with synthetic biology strategies may contribute to a sustainable approach to plastic waste management.

## Data Availability

The datasets presented in this study can be found in online repositories. The names of the repository/repositories and accession number(s) can be found in the article/Supplementary material.

## Footnotes

^1^ https://www.ezbiocloud.net

^2^ http://www.bioinformatics.babraham.ac.uk/projects/fastqc

^3^ https://blast.ncbi.nlm.nih.gov/Blast.cgi

^4^ https://itol.embl.de/

^5^ https://card.mcmaster.ca/

^6^ https://proksee.ca/
