# Supplementary material for: Functional and genomic insights into BHET-degrading Stenotrophomonas sp. isolated from the marine plastisphere
Source: Front Microbiol. 2025 Oct 23;16:1680692. doi: 10.3389/fmicb.2025.1680692 (PMC12588907; doi:10.3389/fmicb.2025.1680692)

**Table S1.** Genome information of *Stenotrophomonas* sp. WED208 and related species in *Stenotrophomonas*.

| **Strain** | **GenBank accession number** | **Genome size** | **Number of contigs** | **Contig L50** | **GC percent** | **Genes (total)** | **CDS (total)** | **rRNAs** | **tRNAs** | **ncRNAs** | **Pseudo Genes (total)** |
| --- | --- | --- | --- | --- | --- | --- | --- | --- | --- | --- | --- |
| *S.* sp. WED208 | JAZARB000000000 | 4.7 | 3 | - | 66.5 | 4,253 | 4,176 | 3 | 73 | - | - |
| *P. hibiscicola* ATCC 19867 | ARNB00000000 | 4.4 | 20 | 4 | 66.5 | 4,086 | 4,009 | 8 | 65 | 4 | 32 |
| *S. acidaminiphila* DSM 13117 | JAMHDZ010000000 | 3.9 | 25 | 3 | 69 | 3,554 | 3,489 | 4 | 57 | 4 | 49 |
| *S. aracearum* A5588 | NZ_CP115543 | 4.3 | 1 | 1 | 66.5 | 3,849 | 3,849 | 10 | 68 | 5 | 42 |
| *S. bentonitica* BIIR7 | JAAZUH000000000 | 4.4 | 191 | 38 | 66.5 | 4,003 | 3,954 | 1 | 44 | 4 | 158 |
| *S. chelatiphaga* DSM 21508 | LDJK00000000 | 4 | 148 | 22 | 67 | 3,524 | 3,378 | 5 | 62 | 1 | 78 |
| *S. cyclobalanopsidis* TPQG1-4 | VYKI00000000 | 4.2 | 97 | 20 | 67 | 3,844 | 3,767 | 7 | 66 | 4 | 82 |
| *S. daejeonensis* JCM 16244 | LDJP00000000 | 3.3 | 124 | 23 | 68.5 | 2,943 | 2,806 | 3 | 56 | 1 | 77 |
| *S. geniculata* JCM 13324 | LLXT00000000 | 4.8 | 170 | 27 | 66 | 4,496 | 4,337 | 8 | 66 | 1 | 84 |
| *S. ginsengisoli* DSM 24757 | LDJM00000000 | 3.4 | 99 | 13 | 66 | 3,106 | 2,889 | 5 | 76 | 1 | 135 |
| *S. humi* DSM 18929 | LDJI00000000 | 4.1 | 92 | 10 | 64 | 3,704 | 3,499 | 5 | 62 | 2 | 136 |
| *S. indicatrix* WS40 | PEJS00000000 | 4.6 | 9 | 1 | 66.5 | 4,171 | 4,095 | 7 | 65 | 4 | 39 |
| *S. koreensis* DSM 17805 | LDJH00000000 | 3 | 58 | 6 | 66 | 2,827 | 2,598 | 6 | 75 | 1 | 147 |
| *S. lactitubi* M15 | PHQX00000000 | 4.9 | 7 | 1 | 66 | 4,521 | 4,444 | 7 | 66 | 4 | 75 |
| *S. maltophilia* ATCC 13637 | MTGD00000000 | 5 | 192 | 24 | 66 | 4,711 | 4,635 | 6 | 66 | 4 | 95 |
| *S. mori* CPCC 101365 | JAIKTS000000000 | 3.4 | 13 | 2 | 70 | 2,957 | 2,891 | 5 | 57 | 4 | 29 |
| *S. muris* DSM 28631 | JANKBX000000000 | 4.7 | 33 | 5 | 66.5 | 4,402 | 4,332 | 3 | 63 | 4 | 30 |
| *S. nematodicola* W5 | WIAY00000000 | 4.4 | 22 | 3 | 67.5 | 4,002 | 3,893 | - | - | - | - |
| *S. nitritireducens* DSM 12575 | LDJG00000000 | 4 | 95 | 9 | 68.5 | 3,534 | 3,362 | 3 | 54 | 1 | 114 |
| *S. oahuensis* A5586 | NZ_CP115541 | 4.7 | 2 | 1 | 65.5 | 4,197 | 4,117 | 7 | 69 | 4 | 20 |
| *S. panacihumi* JCM 16536 | LLXU00000000 | 3.9 | 141 | 22 | 69 | 3,557 | 3,403 | 4 | 51 | 1 | 98 |
| *S. pavanii* DSM 25135 | LDJN00000000 | 4.3 | 129 | 18 | 67.5 | 3,951 | 3,835 | 6 | 67 | 1 | 42 |
| *S. pennii* Sa5BUN4 | JACSQS000000000 | 3.9 | 79 | 14 | 66.5 | 3,535 | 3,464 | 3 | 64 | 4 | 22 |
| *S. pictorum* JCM 9942 | LLXS00000000 | 3.5 | 84 | 13 | 66 | 3,237 | 3,099 | 6 | 61 | 1 | 70 |
| *S. rhizophila* DSM 14405 | CP007597 | 4.6 | 8 | 2 | 67.3 | 4,047 | 3,938 | 12 | 67 | 1 | 29 |
| *S. rhizophila*  QL-P4 | CP016294 | 4.2 | 1 | 1 | 67 | 3,731 | 3,657 | - | - | - | - |
| *S. sepilia* SM16975 T | LXXZ00000000 | 4.6 | 119 | 16 | 66.5 | 4,238 | 4,162 | 5 | 67 | 4 | 69 |
| *S. terrae* DSM 18941 | LDJJ00000000 | 4.4 | 143 | 17 | 64 | 3,841 | 3,645 | 3 | 60 | 1 | 132 |
| *S. tumulicola* JCM 30961 | JACGXS000000000 | 4.3 | 26 | 4 | 65.5 | 3,846 | 3,774 | 6 | 62 | 4 | 31 |
| *S. lacuserhaii* KCTC 82901 | JACHQY010000000 | 4 | 28 | 4 | 66.5 | 3,594 | 3,488 | - | - | - | - |
| *S. riyadhensis* LMG 33162 | JAHWBK010000000 | 5 | 37 | 6 | 66 | 4,612 | 4,491 | 5 | 66 | 4 | 46 |

**Table S2.** Antibiotic-resistant genes identified through Comprehensive Antibiotic Resistance Database.

| **Best Hit Antibiotic Resistance Ontology (ARO)** | **Drug Class** | **Resistance Mechanism** | ***S.* sp. WED 208** |
| --- | --- | --- | --- |
| *smeR* | aminoglycoside antibiotic; cephalosporin; cephamycin; penam | antibiotic efflux | 98.3 |
| *APH(9)-Ic* | aminoglycoside antibiotic | antibiotic inactivation | 95.8 |
| *AAC(6')-Iz* | aminoglycoside antibiotic | antibiotic inactivation | 90.0 |
| *adeF* | fluoroquinolone antibiotic; tetracycline antibiotic | antibiotic efflux | 60.2 |
| *qacJ* | disinfecting agents and antiseptics | antibiotic efflux | 45.7 |

The most abundant mechanisms included genes encoding antibiotic inactivation enzymes (APH and AAC families) and antibiotic target modification proteins (gyrA and rpoB), highlighting their significance in bacterial resistance. Other detected mechanisms included target protection (QnrB family), target replacement (*fabV*), efflux pump systems (e.g., *EmrAB-TolC*) and additional resistance-related genes (*gidB*, *PgsA*, *OprB* and *OxyR*).

**Table S3.** Antimicrobial resistance mechanism based on BV-BRC genome annotation.

| **No.** | **Antimicrobial Resistant Mechanism** | **Genes** |
| --- | --- | --- |
| 1 | Antibiotic inactivation enzyme | *APH(3')-II/APH(3')-XV; AAC(6')-Ic,f,g,h,j,k,l,r-z; L1 family* |
| 2 | Antibiotic target in susceptible species | *Alr, Ddl, dxr, EF-G, EF-Tu, folA, Dfr, folP, gyrA, gyrB, Iso-tRNA, kasA, MurA, rho, rpoB, rpoC, S10p, S12p* |
| 3 | Antibiotic target protection protein | QnrB family |
| 4 | Antibiotic target replacement protein | *fabV* |
| 5 | Efflux pump conferring antibiotic resistance | *EmrAB-OMF, EmrAB-TolC, MacA, MacB, MdtABC-TolC, TolC/OpmH* |
| 6 | Gene conferring resistance via absence | *gidB* |
| 7 | Protein altering cell wall charge conferring antibiotic resistance | *PgsA* |
| 8 | Protein modulating permeability to antibiotic | OprB family |
| 9 | Regulator modulating expression of antibiotic resistance genes | *OxyR* |

**TABLE S4.** *Stenotrophomonas* species with plastic-degrading activity.

| **Strain** | | **Isolation source** | | **Plastic** | | **Treatment & degradation efficiency** | | **Note** | | **Reference** | |
| --- | --- | --- | --- | --- | --- | --- | --- | --- | --- | --- | --- |
| *S. pavanii* JWG-G1 | | refuse landfill | | PET | | pre-treatment | | - Subsequent degradation by Thermobifida fusca cutinase (TfC) | | (Huang et al., 2022) | |
| *S.* sp. | | Cerrado soil | | PE | | unpretreatment | | - Showed metabolic activity and cellular viability after a 90-day incubation with PE as the sole carbon source | | (Peixoto et al., 2017) | |
| *S.* sp. P2 | | municipal waste dumpsite soil | | LDPE | | beads and film, 70% ethanol and UV rays | | - Incubated in a carbon free basal medium with LDPE beads as sole carbon source for 100 days | | (Dey et al., 2020) | |
| *S*. *pavanii* CC18 | | abandoned solid waste dump site | | LDPE | | modified LDPE | | - LDPE films modified with food grade dye sensitized TNPs and starch blend | | (Mehmood et al., 2016) | |
| *S.* sp. AK1 | | solid waste-dumping sites of Faisalabad, Pakistan | | LDPE | | 32% weight loss, 150d | | - Film | | (Nadeem et al., 2021) | |
| *S. maltophilia* XP1 | | urban soil | | PS microplastic particles | | 6.9% weight loss 30 days, 9.5% weight loss 60 days | | - Anhydrous ethanol, oven 50℃ overnight, UV 30min | | (Xiang et al., 2023b) | |
| *S.* sp. | |  | | LDPE | | 10.15% weight loss 3 month | |  | | (Mazaheri and Nazeri, 2024) | |
| *S. maltophilia* S6 | |  | | LDPE | |  | |  | | (Oluwole et al., 2024) | |
| *S. panacihumi* PA3-2 | | soil | | PP powder | | unpretreated, Mw decreased 90d | |  | | (Jeon and Kim, 2016) | |
| *S.* sp. RZS 7 | | plastic contaminated sites | | poly-β-hydroxybutyrate (PHB) | | PHB depolymerase | |  | | (Wani et al., 2016) | |
| *S.* sp. YCJ1 | | farmland soil | | Poly(butylene adipate-co-terephthalate) (PBAT) | | 10.14 wt.% (504 μg/(day•cm2) of PBAT in 5 days | | - Film, biodegradable polymer | | (Jia et al., 2021) | |
| *S. acidaminiphila* BDBP 071 | | tomato rhizosphere soil | | DBP | | Dibutyl phthalate, plasticizer | |  | | (Zhang et al., 2023) | |
| *S*. sp. ZS-S-01 | | agricultural field | | fenvalerate and 3-phenoxybenzoic acid (3-PBA) | | bioremediation | |  | | (Chen et al., 2011) | |
| *S*. *maltophilia* MHF ENV20 | | Surya River, Palghar | | chlorpyrifos | |  | |  | | (Dubey and Fulekar, 2012) | |

**Figure S1.** The expermental flowchart of isolation of associated bacteria with plastisphere in this study.

**Figure S2.** **(A)** Overview of the RAST subsystem categories of strain WED208 genome annotation. **(B)** EggNOG classifications of the annotated strain WED208 genome. The functional annotations were divided into 23 categories.

**Figure S3.** Anvi’o pangenome visualization of nine *Stenotrophomonas* genomes. Genomes are sorted based on the presence/absence of gene clusters. The inner rings 1-9 show the presence (black) or absence of gene clusters in the nine genomes. Rings 10 and 11 show the number of genes in a particular gene cluster and the number of genomes contributing to a particular gene cluster, respectively. The outermost green, orange, and purple represent core, accessory, and singleton genes, respectively.

**Figure S4.** HPLC analysis of PET and TPA degradation by *Stenotrophomonas* sp. WED208 in R2A medium over 30 days. (A) PET without cells (control), (B) PET with WED208, (C) TPA without cells (control), and (D) TPA with WED208.

**Figure S1**


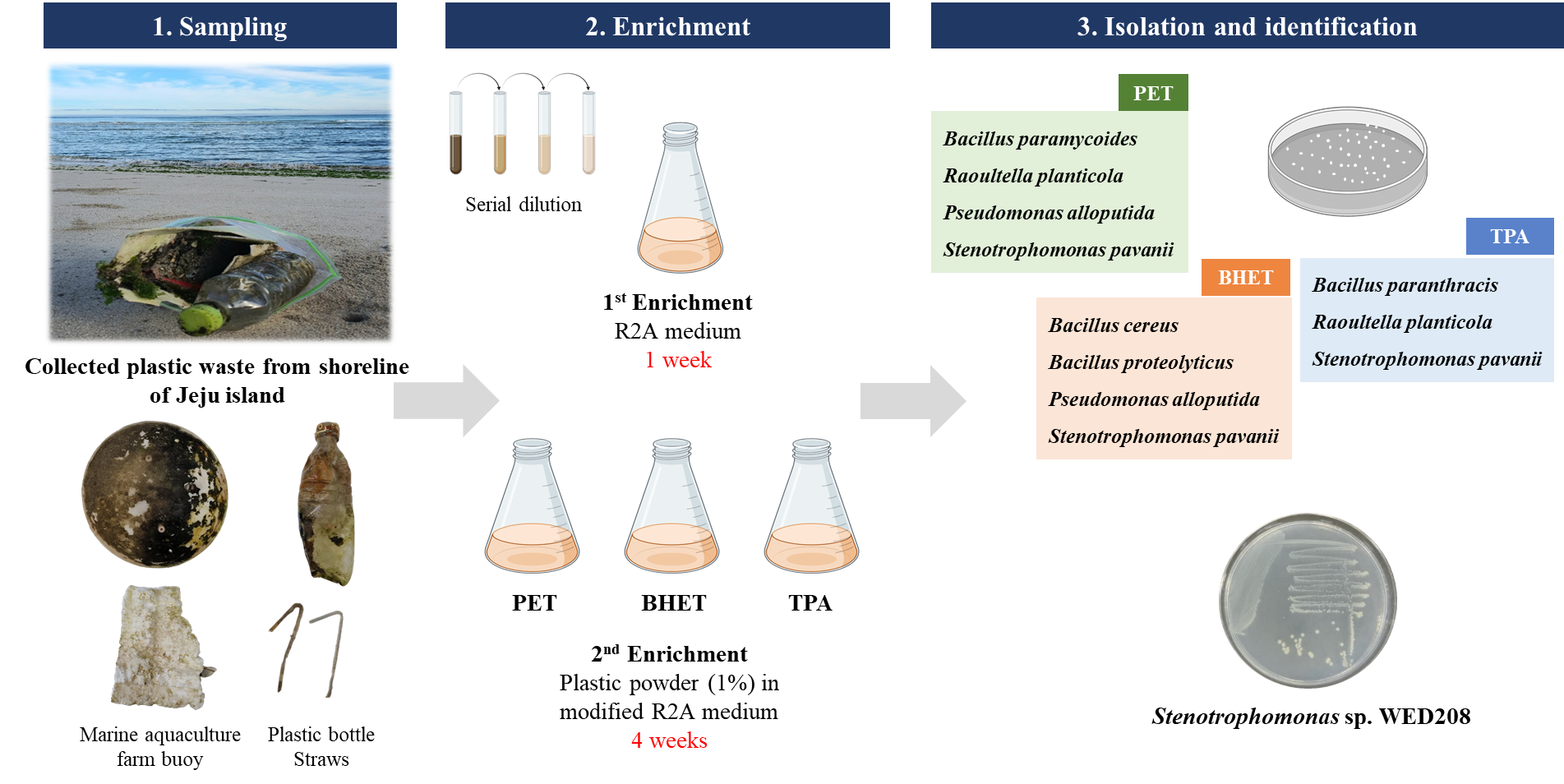


**Figure S2**


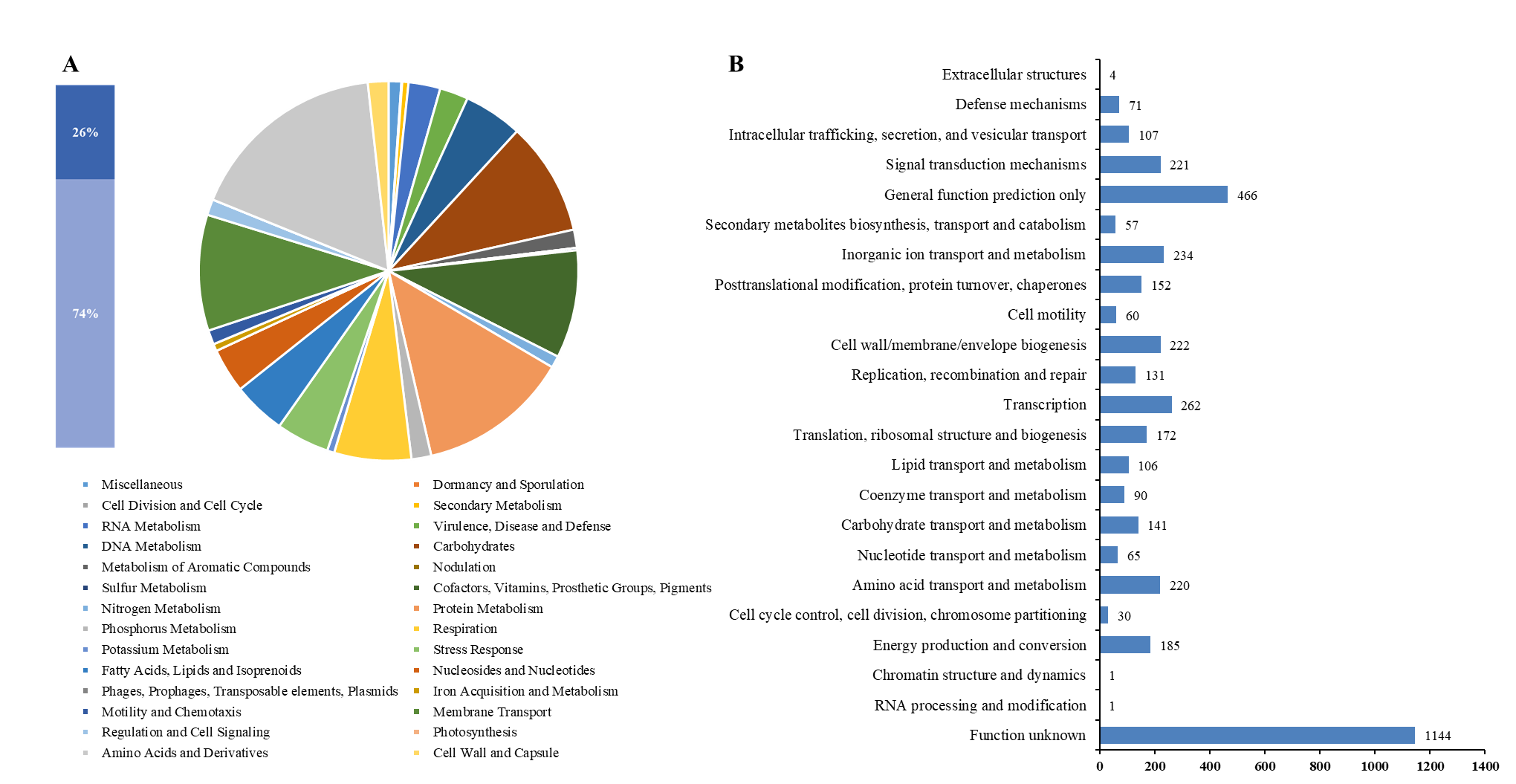


**Figure S3**


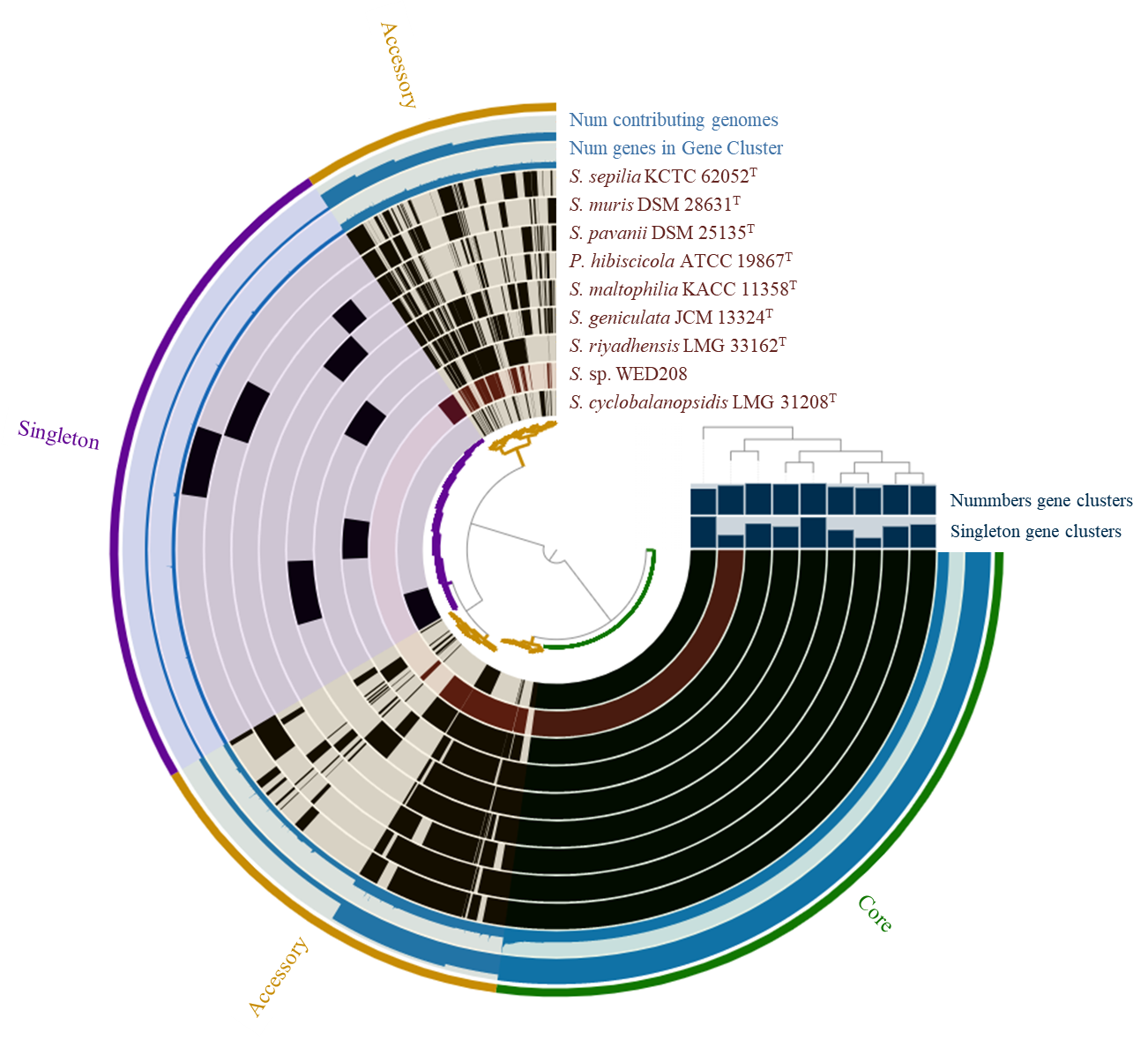


**Figure S4**


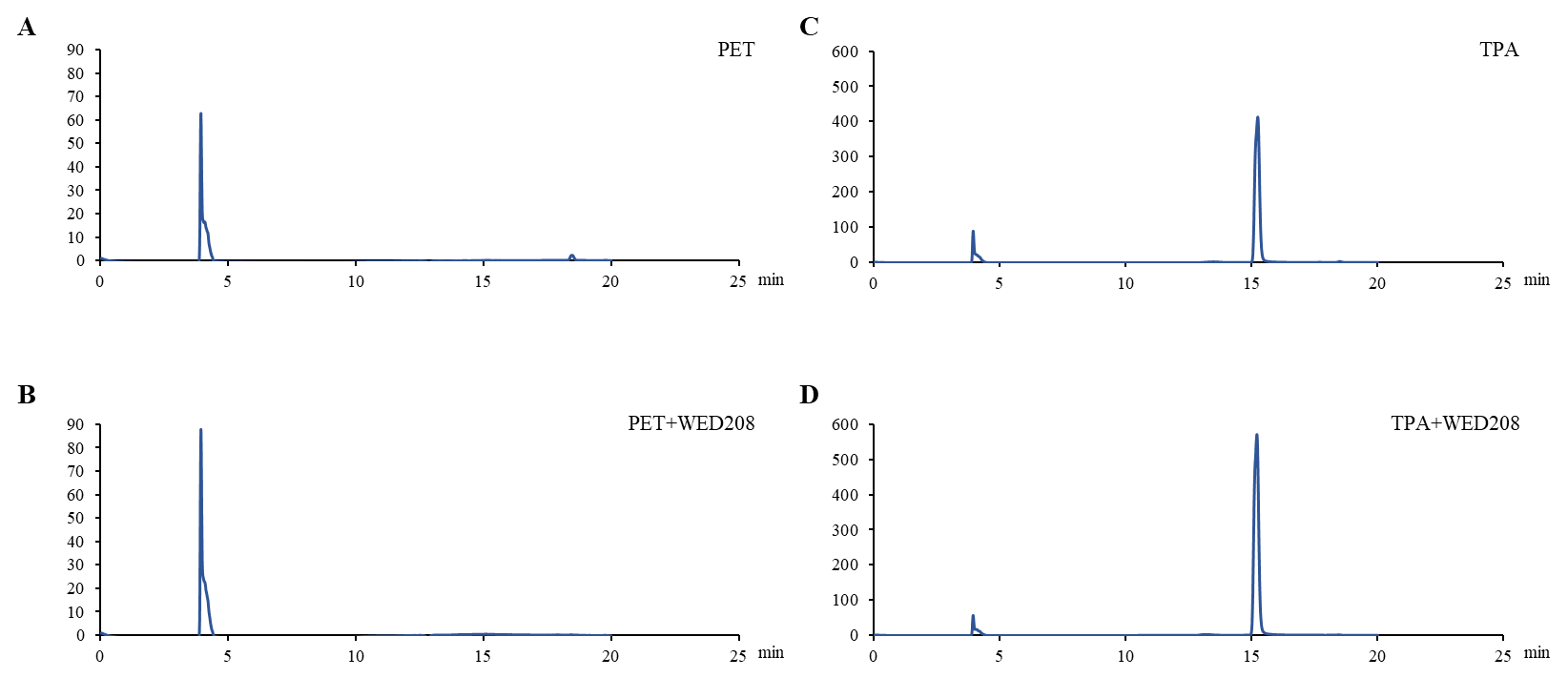

Supplement: Supplementary file 1 [file Supplementary_file_1.docx]
